# Genetic diversity of *Plasmodium falciparum* AMA-1 antigen from the Northeast Indian state of Tripura and comparison with global sequences: implications for vaccine development

**DOI:** 10.1186/s12936-022-04081-1

**Published:** 2022-02-22

**Authors:** Tulika Nirmolia, Md. Atique Ahmed, Vinayagam Sathishkumar, Nilanju P. Sarma, Dibya R. Bhattacharyya, Pradyumna K. Mohapatra, Devendra Bansal, Praveen K. Bharti, Rakesh Sehgal, Jagadish Mahanta, Ali A. Sultan, Kanwar Narain, Saurav J. Patgiri

**Affiliations:** 1 ICMR - Regional Medical Research Centre, North East Region, Dibrugarh, Assam 786001 India; 2 Department of Microbiology and Immunology, Weill Cornell Medicine - Qatar, Cornell University, Doha, Qatar; 3 ICMR - National Institute for Research in Tribal Health, Jabalpur, Madhya Pradesh 482003 India; 4 Department of Medical Parasitology, Postgraduate Institute of Medical Education and Research, Chandigarh, Punjab 160012 India; 5 Present Address: Ministry of Public Health, Doha, Qatar; 6 Present Address: SRL Reference Laboratory, Mumbai, 400060 India

**Keywords:** Apical Membrane Antigen 1 (AMA-1), *Plasmodium falciparum*, Genetic diversity, Tripura, Northeast India

## Abstract

**Background:**

Malaria continues to be a major public health problem in the Northeastern part of India despite the implementation of vector control measures and changes in drug policies. To develop successful vaccines against malaria, it is important to assess the diversity of vaccine candidate antigens in field isolates. This study was done to assess the diversity of *Plasmodium falciparum* AMA-1 vaccine candidate antigen in a malaria-endemic region of Tripura in Northeast India and compare it with previously reported global isolates with a view to assess the feasibility of developing a universal vaccine based on this antigen.

**Methods:**

Patients with fever and malaria-like illness were screened for malaria and *P. falciparum* positive cases were recruited for the current study. The diversity of PfAMA-1 vaccine candidate antigen was evaluated by nested PCR and RFLP. A selected number of samples were sequenced using the Sanger technique.

**Results:**

Among 56 *P. falciparum* positive isolates, *Pfama-1* was successfully amplified in 75% (n = 42) isolates. Allele frequencies of PfAMA-1 antigen were 16.6% (n = 7) for 3D7 allele and 33.3% (n = 14) in both K1 and HB3 alleles. DNA sequencing revealed 13 haplotypes in the *Pfama-1* gene including three unique haplotypes not reported earlier. No unique amino-acid substitutions were found. Global analysis with 2761 sequences revealed 435 haplotypes with a very complex network composition and few clusters. Nucleotide diversity for Tripura (0.02582 ± 0.00160) showed concordance with South-East Asian isolates while recombination parameter (Rm = 8) was lower than previous reports from India. Population genetic structure showed moderate differentiation.

**Conclusions:**

Besides documenting all previously reported allelic forms of the vaccine candidate PfAMA-1 antigen of *P. falciparum*, new haplotypes not reported earlier, were found in Tripura. Neutrality tests indicate that the *Pfama-1* population in Tripura is under balancing selection. This is consistent with global patterns. However, the high haplotype diversity observed in the global *Pfama-1* network analysis indicates that designing a universal vaccine based on this antigen may be difficult. This information adds to the existing database of genetic diversity of field isolates of *P. falciparum* and may be helpful in the development of more effective vaccines against the parasite.

**Supplementary Information:**

The online version contains supplementary material available at 10.1186/s12936-022-04081-1.

## Background

The World Health Organization (WHO) estimates approximately 229 million new malaria cases and 409,000 deaths due to malaria occurring globally in 2019 [1]. Out of this, South-East Asia contributes 3% of the total caseload. In 2019, India’s contribution to total malaria cases and deaths in the South-East Asian region was around 88% and 86% respectively. In South-East Asia, *Plasmodium vivax* is now the major (51.7%) malaria parasite in circulation [1].

On an average, North East India contributes approximately 7% of the total malaria cases in India [2]. Among the eight states of North East India, Tripura is highly malaria endemic where transmission is persistent [3]. Many outbreaks of malaria have been reported in the past few decades from Tripura [4]. In 2014, a severe malaria outbreak occurred in the state with a high morbidity and mortality rate reported mainly from the Dhalai district [5, 6].

Despite active vector control strategies and artemisinin-based combination therapy (ACT) being implemented universally, drug resistant and genetically diverse *Plasmodium falciparum* is spreading continuously across different parts of the world including North East India [7–10]. An effective vaccine is needed, but despite many efforts initiated throughout the last six decades, still no licensed vaccine is available which shows 100% efficacy against the disease. Recently the RTS,S/AS01 vaccine has gone through phase-III efficacy trial which and has shown partial protection (30–50%) against *P. falciparum* malaria in young children [11–15]. RTS,S/AS01 is the only first-generation malaria vaccine and its large-scale pilot implementation has started in April, 2019 in Malawi, Ghana and Kenya [16, 17]. Based on these results, in October 6, 2021 the World Health Organization recommended the widespread use of RTS,S/AS01 malaria vaccine among children living in sub-Saharan Africa and other regions with moderate to high *P. falciparum* malaria transmission [18].

The main obstacle in producing an effective malaria vaccine is the highly polymorphic nature of the parasite and vaccine candidate genes, which allows the parasite to escape host immunity [19]. The erythrocytic stage of the malaria parasite is important in this respect because most vaccine candidate proteins such as the merozoite surface protein (*MSP*) are expressed while invading the RBCs coinciding with clinical disease [20]. However, these vaccine candidate proteins are highly polymorphic in nature, mandating a detailed understanding of their diversity patterns through field studies in different geographical locations [21, 22]. Among the established vaccine candidate antigens of *P. falciparum*, blood-stage antigens like MSPs have traditionally been given more importance; but other antigens like *P. falciparum* apical membrane antigen (PfAMA-1) also hold promise [23].

One of the leading erythrocytic stage vaccine candidate genes of *P. falciparum* is apical membrane antigen-1 (AMA-1). It is 83 kDa in size and expressed in the late schizont stage of the life cycle of malaria parasite [24]. As described previously, this gene can be classified into three major allelic families by PCR-RFLP technique based on the amino acid differences present outside the Hyper Variable Region (HVR) of the gene [25]. Although its function is not yet clear, many studies documented that antibodies against PfAMA-1 raised in rabbits can inhibit the invasion of red blood cells by both homologous and heterologous *P. falciparum* and vaccines based on PfAMA-1 can induce asymptomatic protection [26–29]. Despite its less polymorphic nature, antibodies to this antigen have shown to confer natural protection against the disease [30–32]. Phase-I vaccine trial has also been conducted on malaria-naive volunteers [33]. It was also reported by previous studies that immunization with PfAMA-1 provides protection against malaria in mice and monkeys [34, 35].

Malaria control requires a coordinated approach based on vector control strategies and basic research such as surveillance of parasite genetic diversity and evolution. Very few studies have reported the diversity of *Pfama-1* gene in northeastern as well as in other parts of India [36–39]. The current study was thus conducted to address this void and to analyse the diversity patterns between Tripura and other parts of the world with a view to understand whether a universal vaccine based on this candidate would be feasible in the near future.

## Methods

### Study location

This study was carried out in the sub-divisional hospitals, primary health centers and villages of Dhalai (23.8467° N, 91.9099° E) and North Tripura (24.0797 °N, 92.2630 °E) districts of Tripura state, India in 2015. These districts share borders with Bangladesh in the north and south and more than 70% of the land area is covered by hills and forests. The area experiences hot, humid summers and a prolonged rainy season.

### Study population

Symptomatic patients with body temperature ≥ 37.5 ºC, age > 1 year, without history of anti-malarial drug consumption and no recent history of fever were included in this study. Presence of *P. falciparum* parasite was screened by rapid diagnostic test (RDT) and confirmed by slide microscopy. The study was conducted with approval from the Institutional Human Ethics committee of ICMR-RMRC North East Region (No. RMRC/DIB./IEC Human/ 2012/667) and all protocols were carried out as per the guidelines of the Indian Council of Medical Research (ICMR). Two ml of whole blood was collected from the *P. falciparum* positive patients after obtaining informed written consent.

### Genomic DNA isolation and *Plasmodium* species identification

Genomic DNA extraction was done from whole blood samples using QIAamp DNA blood mini kit as per the manufacturer’s protocol (Qiagen, CA, USA). *Plasmodium* species specific nested PCR was carried out for confirmation of *P. falciparum* as described previously [40]. Extracted DNA was stored in −20 °C for further molecular analysis.

### Nested PCR for ***Pfama-1 gene***

All the PCR protocols and primers were used as previously described with minor modifications [41]. Primary PCR for *Pfama-1 was* performed using 1 µl of genomic DNA and 1 µM of forward and reverse primers in a 10 µl reaction volume containing 5 µl of 2X Promega master mix. Secondary PCR was performed using 2 µl of primary PCR product as template with 1 µM of each primer and 25 µl of 2X Promega master mix in 50 µl reaction volumes. The PCR amplification conditions were: initial denaturation at 95 °C for 5 min followed by (30 cycles for primary PCR and 35 cycles for secondary PCR) denaturation at 95 °C for 2 min, annealing at 52 °C for 30 s, extension at 68 °C for 45 s and a final extension of 5 min at 68 °C. The PCR product of *Pfama-1* was analysed on 2% agarose gel and expected positive amplicon size was 500 bp.

### Restriction fragment length polymorphism analysis of ***Pfama-1***

After successful amplification of the *Pfama-1* gene, the amplified PCR products were subjected to digestion with three restriction enzymes, viz. Mse1, Ssp1 and Sau3A1 (New England Biolabs) as previously described [42]. Three independent digestions were performed with the three restriction enzymes. The digestion mixture contained 0.4 µl restriction enzyme, 2 µl 10X buffer, 10 µl PCR product and volume made up to 20 µl by adding nuclease free water. Digestion was done for 60 min at 37 °C followed by 20 min enzyme inactivation at 65 °C as described by manufacturer’s protocol with minor modifications. The digested products were analysed on 2.5% agarose gel. The respective band sizes for Mse1, Ssp1 and Sau3A1 enzyme digestions were 285 bp (K1), 400 bp (3d7) and 335 bp (HB3), respectively.

### Sequencing

A limited number of samples (17 out of 42 isolates with successful *Pfama-1* amplification) were selected for sanger sequencing. Samples for sequencing were selected randomly since all the 42 samples which showed positive amplification for *Pfama-1* also showed successful restriction digestion with each of the three enzymes. Since the Indian and global database on *Pfama-1* is quite extensive, sequencing was done for a small number of samples representative of the geographical region under consideration. Selected samples were gel purified using Wizard® SV Gel and PCR Cleanup System (Promega) following manufactures’s protocol. The purified products were outsourced to Eurofins Genomic India private limited, Banglore, for both forward and reverse direction Sanger sequencing.

### Sequence polymorphism, phylogenetic and statistical analysis

The sequences were edited in the software Bioedit v7.0.5.3 and aligned in Clustal W [43, 44]. The BLAST similarity searches were done in GenBank database and representative sequences from other parts of the world were downloaded for comparison. The *Plasmodium reichenowi* strain (Accession No. AJ252087) was included as an outgroup for performing the neutrality tests i.e. Fu & Li’s F and D test. DnaSP version 6 was used to calculate various measures of genetic polymorphism such as haplotype diversity (Hd), nucleotide diversity (π), recombination parameters (R) and different neutrality tests [45]. The π value was calculated to estimate step-wise diversity based on a sliding window of 100 bases with a step size of 25 bp. The genetic differentiation among the populations based on fixation index (Fst) was estimated by using Arlequin 3.5 software [46]. Taking *P. reichenowi* strain as an outgroup, dN/dS values were estimated using SNAP v2.1.1 [47]. Haplotype network of global *Pfama-1* haplotypes was constructed with 2761 sequences following the Minimum Spanning Network algorithm using the software PopART [48]. The list of sequences downloaded from the NCBI database and used in the population genetics and Network analysis is provided as a separate file (Additional file 1: Table S1).

## Results

A total of 56 *P. falciparum* PCR positive isolates were included in this study for genetic diversity analysis. Among these, *Pfama-1 was* amplified in 75.0% (n = 42) isolates.

### Allele prevalence among the isolates

In positive samples, all the three previously reported alleles were observed with fragment sizes of 285 bp (K1), 400 bp (3d7) and 335 bp (HB3) after restriction digestion. Among the 42 positive isolates, frequencies of K1 and HB3 allele types were 33.3% (n = 14) each and 3D7 allele type was 16.6% (n = 7), respectively (Table 1). Besides the three allele types, mixed alleles were also observed in three isolates. An additional allele fragment, not previously reported, bearing a size of approximately 355 bp was observed in 83.3% (n = 35) isolates.

**Table 1:** Allele frequency of *Pfama-1* gene in Tripura

Total sample [56]	Allele	Frequency
	K1	33.30% (n = 14)
	3D7	16.60% (n = 7)
*Pfama-1* (n = 42)	HB3	33.30% (n = 14)
	K1+3D7+HB3	2.40% (n = 1)
	K1+3D7	2.40% (n = 1)
	3D7+HB3	4.70% (n = 2)
	HB3+K1	4.70% (n = 2)

### DNA sequencing and sequence polymorphisms

**Fig. 1:**
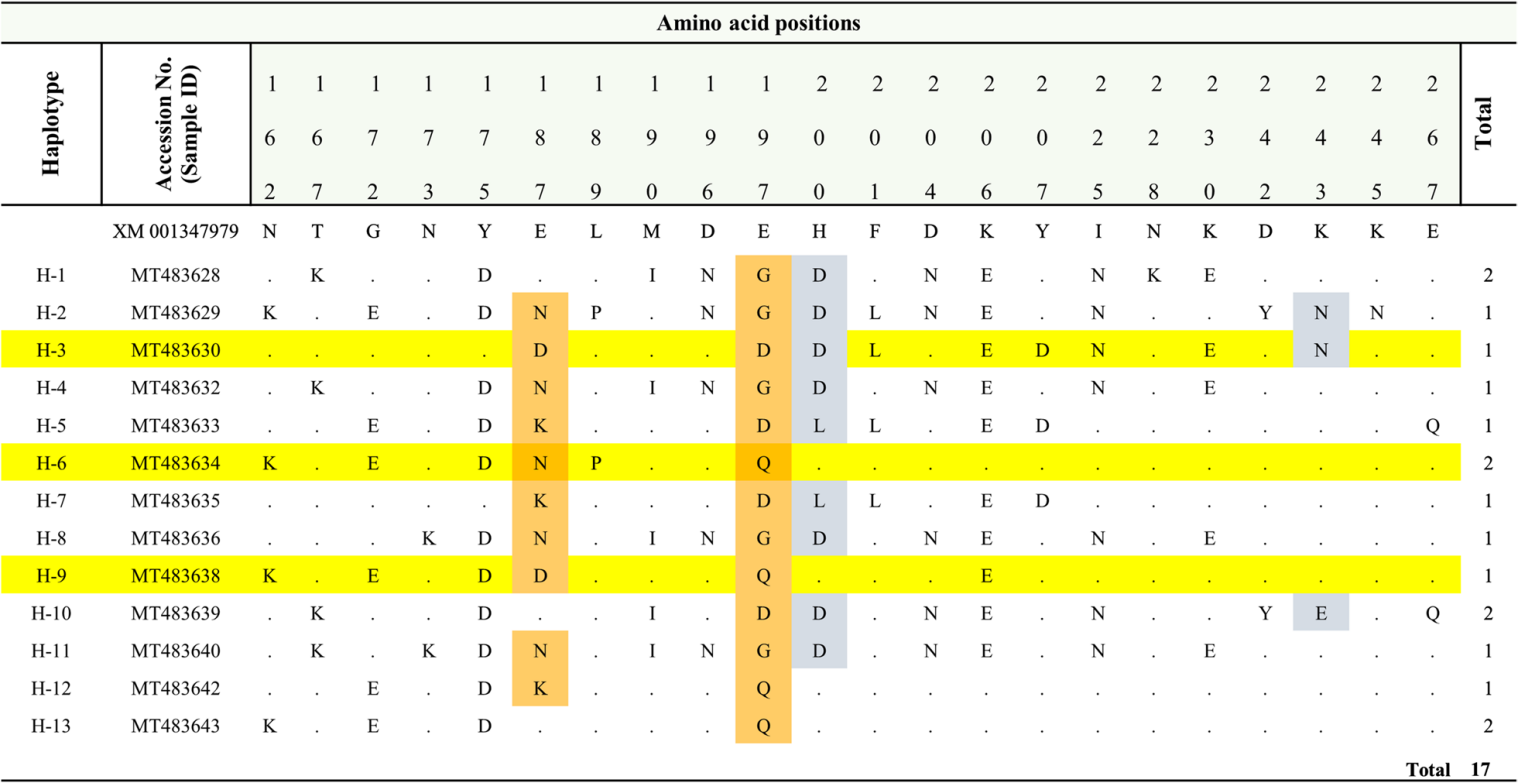
Amino-acid sequence polymorphism observed in Tripura *Pfama-1* isolates. Trimorphic and tetramorphic amino acid are highlighted in blue and orange respectively. New haplotype (H-3, H-6 and H-9) are highlighted in yellow

*Pfama-1* gene was successfully sequenced for a total of 17 *P. falciparum* isolates. The sequences were submitted to the NCBI database with *Accession* numbers: MT483644 to MT483628. In this study, a total number of 13 different haplotypes were observed in *Pfama-1* antigenic gene of Tripura *P. falciparum* isolates (Table 2). Out of these, three unique haplotypes were observed: H-3/MT483630, H6-/MT483634, MT483637 and H-9/MT483638 when compared with 2744 global *Pfama-1* sequences. Further, it was observed that the H-5/MT483633 haplotype was reported only from India, the H-8/MT483636 haplotype was reported earlier from India and Myanmar and the H-7/MT483635 haplotype was reported only from Uganda. Other eight haplotypes were earlier reported from various parts of the world (Table 2). On multiple sequence alignment of the 17 *Pfama-1* nucleotide sequences with the reference strain (XM-001347979), 21 amino acid substitutions were found in domain 1 of *Pfama-1* gene (Fig. 1). Eighteen dimorphic (N162K, T167K, G172E, N173K, Y175D, L189P, M190I, D196N, F201L, D204N, K206E, Y207D, I225N, N228K, K230E, D242Y, K245N, E267Q), two trimorphic (H200D/L, K243N/E) and two tetramorphic (E187N/D/K, E197G/D/Q) substitutions were found.

**Table 2:** *Pfama-1* haplotypes observed in the study and their geographical distribution

Gene	No. of seq. analysed	No. of haplotype	Haplotype (H)	Accession No. (Sample ID)	Reported from
			H-1	MT483628 & MT483631	India, Benin: Cotonou, Ghana, Tanzania, Philippines, Gambia, Mali, Uganda, Kenya, Nigeria, Saudi Arabia
*Pfama-1*	17	13	H-2	MT483629	Uganda, Ghana, Gambia, Mali, Kenya, Nigeria, Saudi Arabia
			H-3	MT483630	This study
			H-4	MT483632	India, Iran, Thailand, Myanmar, Tanzania, Kenya
			H-5	MT483633	India
			H-6	MT483634 & MT383637	This study
			H-7	MT483635	Uganda
			H-8	MT483636	India, Myanmar
			H-9	MT483638	This study
			H-10	MT483639 & MT483641	India, Kenya, Nigeria, Mali, Gambia, Cameroon
			H-11	MT383640	Thailand, Malaysia, Philippines, Solomon Island, Papua New Guinea
			H-12	MT483642	India, Myanmar, Thailand, Solomon Island, Philippines, Kenya, Mali, Tanzania,, Gambia, Papua New Guinea, Saudi Arabia, Uganda
			H-13	MT483643 & MT483644	India, Myanmar, Thailand, China, Mali, Vanuatu, Papua New Guinea

### Haplotype network analysis

**Fig. 2:**
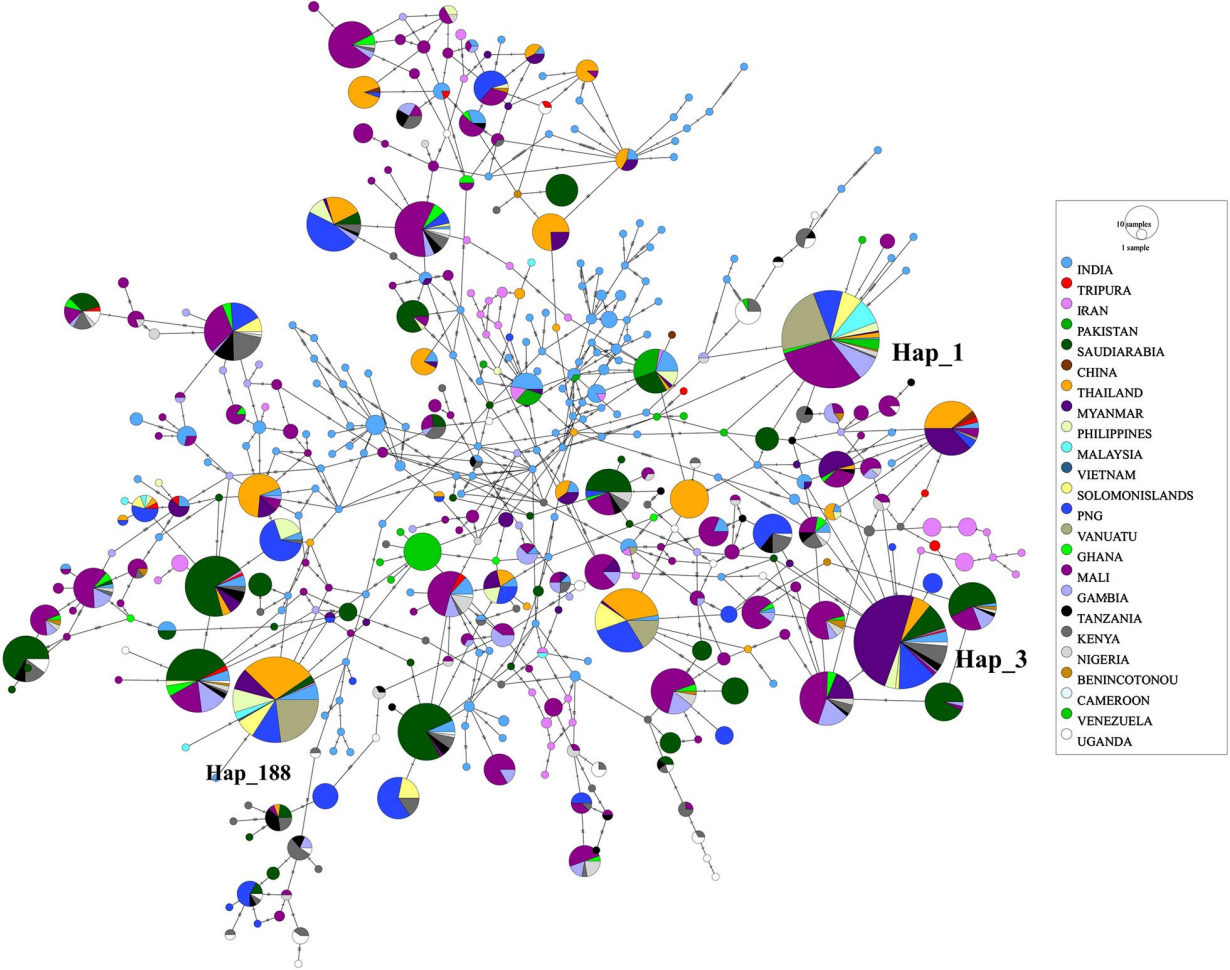
Haplotype network of global *Pfama-1* isolates created using PopArt (Minimum Spanning Network algorithm). Isolates are colour-coded according to the country of origin and the size of the vertex represents the frequency of the haplotype. Hap_1, Hap_3 and Hap_188 are marked separately. Haplotypes from Tripura observed in this study are shown in red

Global analysis with 2761 sequences (including 17 sequences from current study and one reference 3d7 strain) from NCBI database revealed 435 haplotypes out of which 261 (60%) were singletons. Three haplotypes detected in the current study were unique (Hap_299/MT483630, Hap_301_/MT483634, MT483637 and Hap_302/MT483638) and not found amongst the analysed global sequences (Fig. 2). The analysis included sequences from Tripura and 23 malaria endemic countries, including India. From the haplotype network, it was observed that although there was clustering of haplotypes from different countries, the overall picture was complex. Indian isolates in particular appeared to be clustered with a number of global isolates; however, it was observed that there were many unique haplotypes from India not reported earlier from other parts of the world. As many as 169 haplotypes were obtained from the 266 Indian sequences (excluding those from Tripura in the present study) included in the analysis. This was followed by Mali, Kenya and Gambia with 109, 59 and 48 haplotypes each. Three major haplotypes were seen in the Minimum Spanning Network analysis: Hap_1 (176 isolates including the 3d7 reference strain), Hap_3 (161 isolates) and Hap_188 (135 isolates). Hap_1 contained isolates from 15 countries spread across Africa, South America, Oceania and South-East Asia including India; the maximum number of isolates were from Mali (30.68%). Hap_3 included isolates from the Middle East, South and South-East Asia (including Tripura), Africa and Oceania; highest frequency of isolates belonged to Myanmar (49.07%). Hap_188 isolates predominantly belonged to Middle East, South and South-East Asia and Oceania, with no African isolates.

### Nucleotide diversity and natural selection of ***Pfama-1*** isolates from Tripura compared to global isolates

For analysis of nucleotide diversity and natural selection of *Pfama-1* isolates, the sequences from Tripura were compared with other Indian isolates (n = 266) as well as isolates (n = 2485) from twenty other malaria endemic countries spread out across Asia, Middle-East, South America, Africa and Oceania. For the 17 Tripura *P. falciparum* sequences included in this analysis, the calculated nucleotide diversity (π) was 0.02582 and average number of pairwise nucleotide differences (k) was 10.610. When these values were compared with other global *P. falciparum* isolates, it was observed that π value was similar to South-East Asian isolates (Thailand = 0.02565, Philippines = 0.02512). Highest nuclotide diversity was observed in Uganda (π = 0.03187) and lowest was observed among South American (Venezuela= 0.01469) and South-East Asian (Pakistan = 0.01348) isolates. The average number of pairwise nucleotide differences (k) in the isolates included in the analysis ranged from 5.464 in Venezuela to 11.124 in Kenya (Table 3). The Tripura isolates (k = 10.610) had values similar to other Indian isolates (k = 10.847 ) and isolates from Middle East (Saudi Arabia = 10.570) and Africa (Papua New Guinea = 10.158, Ghana = 10.991, Mali = 10.409, Nigeria = 10.475, Benin:Cotonou = 10.773, Gambia = 10.455, Tanzania = 10.731). The neutrality tests in *Pfama-1* gene i.e. Fu and Li’s D & F test were performed considering *P. reichenowi* as an outgroup species. For the Tripura isolates, the observed values for Fu and Li’s D & F test were 1.36492 and 1.58826, respectively, which were not statistically significant (Table 3). Positive Tajima’s D value (1.14014, P > 0.10), though statistically not significant and a positive dN/dS ratio (2.2237), indicated that the *Pfama-1* gene of Tripura *P. falciparum* isolates was under positive selection indicating that there was no evidence of population bottle-necking. However, inclusion of greater number of sequences from Tripura would have increased the accuaracy of these neutrality tests. When possible, Tajima’s D value was calculated for the other global *P. falciparum* isolates and it was found that it was positive for all countries except India (−1.64633, 0.10 > P > 0.05) and China (−0.01883, P > 0.10) (Table 3). This signifies that these isolates may be under negative selection which differs from other isolates of the world that show balancing or positive selection. The negative values as extracted by Fu and Li’s D and F test also supported the negative selection pattern for Indian isolates and the values were statistically significant.

**Table 3:** *Pfama-1* DNA sequence polymorphism and neutrality tests among global *P. falciparum* isolates

Country	S	M	H	Hd ± SD	π ± SD	k	dN/dS	Tajima’s D	Fu & Li’s D	Fu & Li’s F
This study (n = 17)	27	28	13	0.971 ± 0.028	0.02582 ± 0.00160	10.610	2.2237	1.14014 (P > 0.10)	1.36492 (0.10 > P > 0.05)	1.58826 (0.10 > P > 0.05)
India (n = 266)	115	144	169	0.9934 ± 0.0012	0.02916 ± 0.00076	10.847	2.0302	−1.64633 (0.10 > P > 0.05)	−4.50753 (P < 0.02**)	−3.68175 (P < 0.02**)
Thailand (n = 249)	30	34	30	0.923 ± 0.006	0.02565 ± 0.00030	9.542	4.6526	1.99739 (0.10 > P > 0.05)	0.80761 (P > 0.10)	1.65703 (0.10 > P > 0.05)
Myanmar (n = 193)	28	30	31	0.808 ± 0.026	0.01863 ± 0.00116	6.929	4.6364	0.98548 (P > 0.10)	1.1333375 (P > 0.10)	1.31989 (P > 0.10)
The Philippines (n = 55)	28	29	15	0.900 ± 0.019	0.02512 ± 0.00073	9.344	3.2119	1.55177 (0.10 > P > 0.05)	1.26279 (P>0.10)	1.70738 (0.10 > P > 0.05)
Sabah, Malaysia (n = 24)	21	23	7	0.598 ± 0.00315	0.02029 ± 0.00315	7.547	2.6648	0.83231 (P > 0.10)	-0.03391(P > 0.10)	0.27330 (P > 0.10)
Pakistan (n = 20)	12	12	4	0.574 ± 0.090	0.01348 ± 0.00206	5.016	0.9974	1.73344 (0.10 > P > 0.05)	0.55821 (P > 0.10)	1.07022 (P > 0.10)
Iran (n = 61)	27	31	37	0.967 ± 0.011	0.02382 ± 0.00088	8.861	2.581	1.09608 (P > 0.10)	1.39373 (0.10 > P > 0.05)	1.50595 (0.10 > P > 0.05)
Saudi Arabia (n = 379)	33	37	41	0.939 ± 0.004	0.02841 ± 0.00036	10.570	5.2184	2.36102 (P < 0.05**)	2.15242 (P < 0.02**)	2.63450 (P < 0.02**)
Solomon Island (n = 50)	25	26	8	0.838 ± 0.021	0.02659 ± 0.00066	9.893	3.68	2.31227 (P < 0.05**)	1.51749 (0.10 > P > 0.05)	2.20957 (P < 0.05**)
Papua New Guinea (n = 255)	33	40	30	0.943 ± 0.004	0.02731 ± 0.00034	10.158	4.4694	1.57990 (P > 0.10)	0.75309 (P>0.10)	1.27532 (0.10 > P > 0.05)
Vanuatu (n = 85)	20	20	5	0.633 ± 0.028	0.02239 ± 0.00074	8.331	4.9794	3.21750 (P < 0.01**)	1.31415 (P > 0.10)	2.41559 (P < 0.05**)
Venezuela (n = 40)	22	22	10	0.564 ± 0.088	0.01469 ± 0.00247	5.464	1.1437	0.18867 (P > 0.10)	−1.13830 (P > 0.10)	−0.83629 (P > 0.10)
Ghana (n = 38)	30	34	21	0.963 ± 0.013	0.02955 ± 0.00095	10.991	2.8757	1.25547 (P >0.10)	1.45600 (0.10 > P > 0.05)	1.64929 (0.10 > P > 0.05)
Mali (n = 571)	40	48	109	0.9708 ± 0.0023	0.02798 ± n.d.	10.409	4.6211	1.37111 (P > 0.10)	0.18257 (P > 0.10)	0.74883 (P > 0.10)
The Gambia (n = 126)	35	41	48	0.964 ± 0.002	0.028101 ± 0.00060	10.455	3.7438	1.15934 (P > 0.10)	1.26781 (P > 0.10)	1.38885 (P > 0.10)
Tanzania (n = 62)	32	37	32	0.967 ± 0.009	0.02885 ± 0.00078	10.731	3.343	1.19012 (P > 0.10)	2.08797 (P < 0.05**)	2.00956 (P < 0.05**)
Kenya (n = 140)	35	41	59	0.973 ± 0.005	0.02990 ± 0.00047	11.124	3.8974	1.50064 (P > 0.10)	1.25346 (P > 0.10)	1.53807 (0.10 > P > 0.05)
Nigeria (n = 51)	32	36	31	0.975 ± 0.009	0.02816 ± 0.00104	10.475	3.1601	1.04100 (P > 0.10)	1.49382 (0.10 > P > 0.05)	1.52912 (0.10 > P > 0.05)
Benin: Cotonou (n = 23)	27	28	11	0.985 ± 0.040	0.02896 ± 0.00344	10.773	2.0625	0.72567 (P > 0.10)	0.96969 (P > 0.10)	1.04902 (P > 0.10)
China (n = 4)	15	15	3	0.833 ± 0.222	0.02195 ± 0.00626	8.167	0.8939	−0.01883 (P > 0.10)	1.21504 (P > 0.10)	1.15570 (P > 0.10)
Uganda (n = 59)	33	38	36	0.971 ± 0.011	0.03187 ± 0.00076	11.856	3.8062	1.49092 (P > 0.10)	2.09630 (P < 0.02**)	2.18332 (P < 0.02**)

n, number of sequences included; S, Segregating site; M, Total mutation; H, Haplotype; Hd, Haplotype diversity; π, Nucleotide diversity; k, Average no. of pair wise nucleotide differences; dN/dS values were calculated including *P. reichenowi* as outgroup (Accession No. AJ252087) dN, rate of non-synonymous mutations; dS, rate of synonymous mutations; Fu & Li’s D and F test were performed including *P. reichenowi* as Outgroup (Accession No. AJ252087); Sliding Window option: window length: 100, Step size: 25; Tajima’s D, Fu & Li’s D and F test (P**) = statistically significant, P < 0.05

### Recombination parameter

The minimum number of recombination events (Rm) was calculated along with R values of adjacent sites (Ra) and per gene (Rb). The estimated Rm and R values observed in this study were 8 (Rm), 0.0954 (Ra) and 39.1 (Rb), respectively. When compared with other global Rm and R values, it was observed that the Rm value of the present study was similar to South-East Asia (Thailand = 8, Myanmar = 8) and African countries (Benin: Cotonou = 8). However, previously reported *Pfama-1* isolates from India showed the highest Rm values (Rm = 24), which is in contrast to this study. Similarly, Ra values observed in this study were similar to those from Saudi Arabia, Papua New Guinea, Ghana and Tanzania. Highest Ra values were observed in India, Nigeria and Uganda. Rb values from Tripura showed similarity with isolates from Iran and the Philippines. Highest Rb values amongst the analysed samples were seen from African countries (Ghana, Mali, Gambia, Tanzania, Kenya, Nigeria and Benin: Cotonou) (Table 4).

**Table 4:** Recombination events of *Pfama-1* gene among global *P. falciparum* isolates

Country	Rm	Ra	Rb
This study	8	0.0954	39.1
India	24	0.1864	79.8
Thailand	8	0.0445	57.9
Myanmar	8	0.0074	10
The Philippines	6	0.0218	40.8
Sabah, Malaysia	5	0.0001	0.2
Pakistan	0	0	0.001
Iran	12	0.074	39
Saudi Arabia	13	0.0857	54
Solomon Island	5	0.012	22.5
Papua New Guinea	9	0.1094	63
Vanuatu	5	0.001	1.8
Venezuela	1	0	0.001
Ghana	10	0.0958	179
Mali	12	0.1131	137
The Gambia	12	0.082	146
Tanzania	9	0.0915	171
Kenya	13	0.1171	153
Nigeria	10	0.158	207
Benin: Cotonou	8	0.0755	119
China	0	0.0581	29.3
Uganda	12	0.1822	67.6

Rm, Minimum number of recombination events between adjacent sites; Ra, Recombination parameter between adjacent sites; Rb, Recombination parameter per gene

#### Inter-population differentiation

Fst values were evaluated to estimate the genetic differentiation among global *Pfama-1* populations (Additional file 2:  Table S2). Among all the global isolates, lowest Fst value was observed between Kenya and Uganda (0.00479, p < 0.05) and highest Fst value was observed between China and Venezuela (0.44783, p = 0). When Fst values were compared for Tripura isolates, lowest difference was with Tanzania (0.01168, p > 0.05) and highest difference was with Venezuela (0.24724, p = 0). Low genetic difference of Tripura isolates was also observed with the earlier reported Indian isolates as well as other South-East Asian countries like Thailand, Malaysia, Philippines; African countries like Ghana, Uganda, Benin, Gambia, Kenya and other countries like Saudi Arabia, Mali and PNG, etc. Moderate genetic difference was seen with the isolates of Iran, China and Myanmar. Highest genetic difference was seen with the isolates of Pakistan and Venezuela. However, there was one negative Fst value which was seen between the isolates of Kenya and Tanzania (−0.00052, p > 0.05); this may be due to geographical proximity or inclusion of short sequences in the analysis.

## Discussion

The WHO South-East Asia region comprises of eleven countries and is home to more than a quarter of the world’s population. Nine of these eleven countries are endemic for malaria with three countries, including India, contributing to over 99.5% of the total caseload in the region [1]. In India, there are several hotspots of malaria across the country with diverse epidemiological, ecological and geographical settings. Vector and parasite species also vary depending on the region. Northeast India is a very peculiar setting wherein forest malaria predominates; the region also shares a huge international border and has traditionally acted as a gateway for the spread of drug resistant *Plasmodium* strains from surrounding countries to the Indian mainland [4, 49, 50]. Although malaria has reduced drastically in this region over the years, further reduction and control has been difficult. Anti-malarial drug resistance monitoring and surveillance of parasite genetic diversity and evolution are important molecular tools to understand and minimize the spread of malaria in this region. Currently, Tripura and Mizoram are the two states from this region reporting a substantial number of malaria cases [2]. Many areas in Tripura are endemic for malaria and not much data on diversity of *P. falciparum* vaccine candidate genes like *Pfama-1* is available on field isolates. The current study was done to assess the diversity of *Pfama-1* population circulating in the region through RFLP and Sanger sequencing with a view to compare it with other Indian and global isolates.

Restriction fragment length polymorphism (RFLP) analysis of *Pfama-1* gene showed the presence of all the three reported allelic variants; i.e. K1, 3D7 and HB3 type in the study population with a frequency of 33.30%, 16.60% and 33.30% respectively. In addition to this, 2.4% mixed allele type was observed among the *P. falciparum* isolates and 83.3% (355 bp) isolates did not fit into any of these previously reported groups. Earlier studies among Indian *P. falciparum* isolates have reported mostly point mutations; suggesting that PCR-RFLP based genotyping for *Pfama-1* allele needs further validation since it is inherently incapable of detecting SNPs [37]. The current study has shown a lower frequency of the 3D7 allele as compared to K1 and HB3 allelic variants, which is similar to previous studies from South-East Iran, East Africa, Western and Central Africa [30, 41, 51]. However, many other studies based on SNPs have reported varying frequencies of *Pfama-1* haplotype in different geographical regions [22, 52–54]. *Kang et al.* also reported close similarities between the *Pfama-1* gene in Thailand and Myanmar [53]. Polymorphism in *Pfama-1* gene is not evenly distributed; antigenic diversity of *Pfama-1* between and among global isolates are limited and also it is relatively less polymorphic in nature than the laboratory isolates, possibly due to the positive natural selection and genetic recombination [30, 37, 53, 54]. In high disease transmission areas, allelic diversity of *Pfama-1* is usually very high compared to low transmission areas and in major endemic areas, *Pfama-1* alleles have been found to exhibit similar diversity patterns regardless of geographical region [39, 52, 55].

Sanger sequencing of a few *Pfama-1* isolates revealed thirteen haplotypes in total out of which three haplotypes were unique as depicted in the haplotype network constructed using 2761 global *Pfama-1* sequences (Fig. 2). The haplotype network was dense with many complex connections and no clear clustering was apparent. As many as 435 haplotypes were identified out of which 60% were singletons; India being a major contributor to these low frequency haplotypes. There was no haplotype that was present universally in all the malaria endemic countries included in the analysis. Three haplotypes with sizeable number of isolates from different countries were obtained out of which Hap_1, which included the *P. falciparum* 3D7 strain, formed the biggest cluster with 176 isolates (6.37% of analysed strains). Most of the PfAMA-1 antigen-based vaccines that are currently being evaluated for field use have been designed on the basis of *P. falciparum* 3D7 strain. The current analysis revealed that only a minority of the global isolates belonged to this haplotype (Hap_1); additionally, only one isolate from India and none from Tripura were identical to this haplotype. Studies conducted in Myanmar and Bioko islands have also observed similar haplotype networks with no consistent clustering; although, the number of isolates included in the network analysis was much smaller [53, 56]. In Myanmar, haplotype network constructed using 517 global *Pfama-1* isolates revealed 174 haplotypes and in a separate study conducted in Bioko islands, 296 haplotypes were obtained from the analysis of 790 sequences [53, 56].

Nucleotide diversity figures observed in this study (π = 0.02582 ± 0.00160) were found to be similar to South-East Asian and African isolates and slightly lower than those reported earlier from India (Table 3). However, studies conducted in nearby Indian states like Assam and Orissa have reported similar values [36]. Even Andaman & Nicobar Islands, an Indian union territory and archipelago in the Bay of Bengal, has reported values for nucleotide diversity (0.0226 ± 0.0008) similar to the Tripura isolates [36]. Another study from India has also reported similar nucleotide diversity patterns from Assam, Orissa and North India [39]. Haplotype diversity and average number of pairwise nucleotide differences (k) were also found to be similar in Tripura, Assam, Orissa and Andaman & Nicobar Islands [36]. Haplotype diversity of domain-I of Tripura *Pfama-1* strains was found to be higher than that reported earlier from South-East Asian countries such as Myanmar and Thailand and comparable to those observed in African countries like Ghana and Tanzania [53, 56]. A positive Tajima’s D value for majority of global isolates (except India and China, Table 3) also indicates that the domain-1 of *Pfama-1* is under positive natural selection; this has been reported earlier in several studies [36, 53, 56]. Negative values for Tajima’s D, which signify negative selection, have been reported earlier from India in Kolkata [39]. A possible explanation for this may be the high number of low frequency haplotypes (as many as 60% of the Indian haplotypes were singletons) observed in the 266 *Pfama-1* sequences included in the analysis. Nucleotide diversity, haplotype diversity and recombination events (*Rm*) were also highest among the Indian isolates (π = 0.02916 ± 0.00076, *Hd* = 0.9934 ± 0.0012, *Rm* = 24), which signifies that the parasite population must have undergone recent expansion with recombination events generating newer alleles.

In the current study, no unique amino-acid substitutions were observed; all amino-acid substitutions reported here have been observed previously in Tanzania, Ghana, Thailand, Bioko island, Pakistan and some other parts of the world [53, 56, 57]. Of these, the N228K mutation encountered in the current study has been found to be more common in African countries unlike Myanmar and Thailand [53]. Although there are differences in the frequency of these amino-acid substitutions across different geographical regions, the overall distribution is largely the same; however, inclusion of larger number of samples from diverse geographical regions of North East India would have provided a more comprehensive picture.

Genetic differentiation among global *Pfama-1* populations was evaluated by estimating the pairwise Fst values and classified as previously described [53]. Tripura isolates exhibited low levels of genetic differentiation when compared to African (Uganda, Tanzania, Nigeria, Mali, Kenya, Ghana, Gambia and Benin: Cotonou) countries. Moderate levels of differentiation were observed with previously reported Indian isolates (0.05351) and isolates from South-East Asia (Myanmar, Malaysia), Iran, China and Vanuatu. High-level differentiation was observed when the Tripura isolates were compared with those of Venezuela (0.24724) and Pakistan (0.22606). This trend is more or less similar to that shown by other Indian isolates when compared globally: high differentiation with Venezuela (0.16519) and Iran (0.15053); moderate differentiation with Vanuatu, Tanzania, Solomon Islands, Pakistan, Myanmar, Malaysia, China and Benin: Cotonou; and low differentiation with all other countries (predominantly African) included in the analysis. Interestingly, it was observed that isolates from Venezuela, the only South American country included in the analysis, showed a high level of genetic differentiation with all other countries. This might be due to the geographical barriers to gene flow among the parasite populations of Venezuela and the other countries (which belong to different continents) analysed. A similar finding is also observed in a study conducted in Bioko Islands where the Venezuelan strains showed high genetic differentiation with nine countries from Africa, South-East Asia and Oceania [56]. Other studies have found moderate levels of differentiation (0.05−0.15) among global *Pfama-1* sequences [53]. Differences in the number of sequences and the country of origin analysed may sometimes lead to variations in the absolute Fst values; however, the overall picture for *Pfama-1* appears to be one of low to moderate genetic differentiation with a few exceptions, which can be explained based on the isolation by distance model. Overall, nucleotide and haplotype diversity figures, haplotype network and inter-population Fst values indicate that a common vaccine design may be complicated.

Limitations of the current study include sequencing of a limited number of samples for the target gene. Additionally, the samples were subjected to both way Sanger sequencing without cloning. While most traditional studies on *Pfama-1* have used PCR-RFLP, we have also used Sanger sequencing, which is one of the strengths of this study. Moreover, no previous study has compared the global *Pfama-1* scenario on such a huge scale.

## Conclusions

The study aimed to evaluate the diversity of PfAMA-1, a less commonly studied vaccine candidate antigen of *P. falciparum* from Tripura, a malaria endemic state in Northeast India. All the three allelic families were observed and neutrality tests indicate that the *Pfama-1* population in Tripura is under balancing selection. This is consistent with global patterns. However, the high haplotype diversity observed in the global *Pfama-1* network analysis indicates that designing a universal vaccine based on this antigen may be difficult. A moderate degree of clustering of isolates from different geographical regions indicate that region specific vaccines based on the PfAMA-1 antigen may have some hope for malaria control.

## Supplementary Information

**Additional file 1:** List of Sequences used in phylogenetic and Haplotype network analysis of *Pfama-1* gene.

**Additional file 2:** Pair wise Fst values of global *Pfama-1* gene.

## Data Availability

All sequences generated in this study are available in the NCBI database (https://www.ncbi.nlm.nih.gov/) and with the corresponding author. The list of additional sequences used in the analysis is available in the Supplementary materials.

## Abbreviations

AMA-1: Apical Membrane Antigen 1
PCR: Polymerase chain reaction
RFLP: Restriction fragment length polymorphism
WHO: World Health Organization
NVBDCP: National Vector Borne Disease Control Programme
ACT: Artemisinin-based combination therapy
RDT: Rapid diagnostic test
