# Supplementary material for: Genetic diversity of Plasmodium falciparum AMA-1 antigen from the Northeast Indian state of Tripura and comparison with global sequences: implications for vaccine development
Source: Malar J. 2022 Feb 22;21:62. doi: 10.1186/s12936-022-04081-1 (PMC8861999; doi:10.1186/s12936-022-04081-1)
Supplement: Supplementary file 1 — Additional file 1: List of Sequences used in phylogenetic and Haplotype network analysis of Pfama-1 gene. [file 12936_2022_4081_MOESM1_ESM.docx]

**Additional Table S1**: List of Sequences used in phylogenetic and Haplotype network analysis of *Pfama-1* gene.

| **Continent** | **Country** | **NCBI accession no.** | **Reference** | **Remark** |
| --- | --- | --- | --- | --- |
| Middle East and South East | This study (n=17) | MT483628-MT483644 |  |  |
|  | India (n=266) |  |  |  |
|  | (Assam, Orissa A&N, Goa, UP) (n=83) | EF413088-EF413170 | 35 |  |
|  | Rajasthan (n=8) | EF543164-EF543168; | 36 |  |
|  |  | EF543174-EF543176 |  |  |
|  | West Bengal (n=5) | EF543169-EF543173 |  |  |
|  | Delhi (n=5) | AY016428, AY016431, AY016434 | 30 |  |
|  |  | AY016437, AY016439 |  |  |
|  | Kolkata (n=100) | KC476551-KC476650 | 38 |  |
|  | Madhya Pradesh (n=63) | HM568725 - HM568787 | Sharma Y. D. | Unpublished data |
|  | Source: NIMR, Delhi (n=2) | DQ455557, DQ455558 | Lalitha P. V. |  |
|  | Iran (n=61 ) | HQ658150-HQ658159 | 22 |  |
|  |  | HM776795-HM776834 |  | excluded: HM776804, HM776821 (very short sequences) |
|  |  | KC413989-KC413999 | Mehrizi et al 2013 |  |
|  | Pakistan (n=20) | MH028193-MH028212 | Irfan M. et al 2018 | Unpublished data |
|  | Saudi Arabia (n=379) | KU863156-KU863534 | Al-Qahtani et al 2016 |  |
|  | China (n=4) | AF277003 | Shan,Z.X.2000 | Unpublished data |
|  |  | KM016428 | Zhou,Y. 2014 |  |
|  |  | U33275 | Zhang et al 1995 |  |
|  |  | DQ174774 | Miao et al 2006 |  |
| Southeast Asia | Thailand (n=249) | MF598898-MF598962 | Lumkul et al 2018 |  |
|  |  | AB715735-AB715814 | Tanabe et al 2013 |  |
|  |  | AB827675-AB827706 | Tanabe et al 2015 |  |
|  |  | AJ494866-AJ494915 | Sawaswong et al 2015 |  |
|  |  | EU86500-EU86507, | Duan et al 2008 |  |
|  |  | EU586477-EU586481 |  |  |
|  |  | EU586375-EU586377, EU586449 |  |  |
|  |  | AY016430, AY016429 | 30 |  |
|  |  | AY016435, AY016436 |  |  |
|  | Myanmar (n=192 ) | KU893276-KU893333 | Kang et al 2018 |  |
|  |  | KT897327-KT897378  KT897380-KT897461 | Zhu et al 2016 |  |
|  | The Philippines (n=55) | AB715815-AB715869 | Tanabe et al 2013 |  |
|  | Sabah, Malaysia (n=24) | KM061758- KM061781 | Lau and Chua, 2014 | Unpublished data |
|  | Vietnam (n=1) | AJ277646 | Kocken et al 2002 |  |
| Oceania | Solomon Island (n=50) | AB715960-AB716009 | Tanabe et al 2013 |  |
|  | Papua New Guinea (n=255) | KF698984 - KF699059 | Arnotte et al 2014 |  |
|  |  | AJ490528-AJ490695 | Cortes et al 2003 |  |
|  |  | EU586358, EU586359, EU586379 | Duan et al 2008 |  |
|  |  | EU586489-EU586496 |  |  |
|  | Vanuatu (n=85) | AB716010-AB716094 | Tanabe et al 2013 |  |
| South America | Venezuela (n=40) | AY016412, AY016414-AY016422 | 30 |  |
|  |  | EU332414-EU332443 | Ord et al 2008 |  |
| Africa | Ghana (n=38 ) | AB715698-AB715734 | Tanabe et al 2013 |  |
|  |  | EU586373, EU586374, EU586391 | Duan et al 2008 |  |
|  | Mali (n=571) | FJ898536-FJ899041 | Takala et al 2009 |  |
|  |  | EU586402-25 |  |  |
|  |  | EU586428-39, EU586444 | Duan et al 2008 |  |
|  |  | EU586450-EU586475 |  |  |
|  |  | EU586446, EU586498 |  |  |
|  | The Gambia (n=126) | FJ555752–FJ555865 | Tetteh et al 2009 |  |
|  |  | EU586360, EU586369 | Duan et al 2008 |  |
|  |  | EU586362-EU586367 |  |  |
|  |  | EU586372, EU586394 |  |  |
|  |  | EU586443, EU586447 |  |  |
|  | Tanzania (n=62) | AB715636-AB715697 | Tanabe et al 2013 |  |
|  | Kenya (n=140) | FN869569-FN869697 | Osier et al 2010 |  |
|  |  | EU586440- EU586442 | Duan et al 2008 |  |
|  |  | AY016413, AY016438 |  |  |
|  |  | AY016432-AY016433 | 30 |  |
|  |  | AY016426-AY016427 |  |  |
|  |  | AY016423-AY016424 |  |  |
|  | Nigeria (n=51) | AJ408300-AJ408350 | Polly et al 2012 |  |
|  | Benin:Cotonou (n=12 ) | AJ271169-AJ271171  AJ271173, AJ271174  AJ271176, AJ271183  AJ271179-AJ271181 AJ271187, AJ271190 |  |  |
|  | Cameroon (n=1) | KM016424 | Zhou, Y. 2014 | Unpublished data |
|  | Uganda (n=59) | LC157527-LC157585 | Ntege et al 2016 |  |
