# Supplementary material for: Genetic diversity of Plasmodium falciparum AMA-1 antigen from the Northeast Indian state of Tripura and comparison with global sequences: implications for vaccine development
Source: Malar J. 2022 Feb 22;21:62. doi: 10.1186/s12936-022-04081-1 (PMC8861999; doi:10.1186/s12936-022-04081-1)
Supplement: Supplementary file 2 — Additional file 2: Pair wise Fst values of global Pfama-1 gene. [file 12936_2022_4081_MOESM2_ESM.docx]

|  | Tripura | Venezuela | Vanuatu | Uganda | Thailand | Tanzania | Solomon | Saudi Arabia | Philippines | PNG | Pakistan | Nigeria | Myanmar | Mali | Malaysia | Kenya | Iran | Ghana | Gambia | China | Benin | India |
| --- | --- | --- | --- | --- | --- | --- | --- | --- | --- | --- | --- | --- | --- | --- | --- | --- | --- | --- | --- | --- | --- | --- |
|  |  |  |  |  |  |  |  |  |  |  |  |  |  |  |  |  |  |  |  |  |  |  |
| Tripura |  | + | + | - | - | - | - | - | - | - | + | - | + | - | + | - | + | - | - | - | - | + |
| Venezuela | 0.24724 |  | + | + | + | + | + | + | + | + | + | + | + | + | + | + | + | + | + | + | + | + |
| Vanuatu | 0.08364 | 0.2111 |  | + | + | + | - | + | + | + | + | + | + | + | - | + | + | + | + | - | + | + |
| Uganda | 0.03024 | 0.2265 | 0.0874 |  | + | - | + | + | + | + | + | - | + | + | + | - | + | - | + | - | - | + |
| Thailand | 0.04683 | 0.21503 | 0.09104 | 0.0481 |  | + | + | + | + | + | + | + | + | + | + | + | + | + | + | - | - | + |
| Tanzania | **0.01168** | 0.25093 | 0.11931 | 0.01281 | 0.04361 |  | + | + | + | - | + | - | + | - | + | - | + | - | - | - | - | + |
| Solomon | 0.03118 | 0.1898 | 0.0421 | 0.04614 | 0.04613 | 0.04838 |  | + | + | + | + | + | + | + | + | + | + | + | + | - | - | + |
| Saudi Arabia | 0.0274 | 0.17452 | 0.10014 | 0.04074 | 0.06097 | 0.02738 | 0.03393 |  | + | + | + | + | + | + | + | + | + | + | + | - | + | + |
| Philippines | 0.03578 | 0.18151 | 0.07287 | 0.05546 | 0.04516 | 0.05616 | 0.03496 | 0.0317 |  | + | + | + | + | + | + | + | + | + | + | - | + | + |
| PNG | 0.01716 | 0.19127 | 0.07703 | 0.02616 | 0.01792 | 0.01182 | 0.02153 | 0.0311 | 0.03352 |  | + | - | + | + | + | + | + | - | + | - | - | + |
| Pakistan | 0.22606 | 0.36778 | 0.27325 | 0.16271 | 0.11664 | 0.16813 | 0.21576 | 0.1897 | 0.17392 | 0.13619 |  | + | + | + | + | + | + | + | + | - | + | + |
| Nigeria | 0.03322 | 0.2164 | 0.11082 | 0.0176 | 0.04848 | 0.0133 | 0.03994 | 0.0211 | 0.0509 | 0.01835 | 0.17593 |  | + | + | + | - | + | - | - | - | - | + |
| Myanmar | 0.12116 | 0.33962 | 0.1551 | 0.12942 | 0.11705 | 0.11484 | 0.16036 | 0.1591 | 0.18239 | 0.09086 | 0.24005 | 0.14337 |  | + | + | + | + | + | + | - | - | + |
| Mali | 0.02535 | 0.21522 | 0.09556 | 0.0205 | 0.02373 | 0.00992 | 0.04891 | 0.0481 | 0.05749 | 0.01278 | 0.12251 | 0.01852 | 0.07713 |  | + | + | + | - | - | - | - | + |
| Malaysia | 0.1117 | 0.32515 | 0.02501 | 0.11473 | 0.1499 | 0.15264 | 0.0994 | 0.1503 | 0.13699 | 0.12355 | 0.38257 | 0.15873 | 0.17828 | 0.13062 |  | + | + | + | + | + | - | + |
| Kenya | 0.0201 | 0.21396 | 0.10474 | **0.00479** | 0.03984 | **-0.00052** | 0.0405 | 0.0251 | 0.04648 | 0.01409 | 0.1459 | 0.00779 | 0.12276 | 0.0125 | 0.14156 |  | + | - | - | - | - | + |
| Iran | 0.13294 | 0.3522 | 0.19703 | 0.13523 | 0.13657 | 0.12925 | 0.15599 | 0.1413 | 0.14931 | 0.11971 | 0.26504 | 0.14436 | 0.18215 | 0.12412 | 0.23838 | 0.12613 |  | + | + | + | + | + |
| Ghana | 0.0196 | 0.26079 | 0.12739 | 0.01839 | 0.02723 | 0.00652 | 0.0527 | 0.0304 | 0.04103 | 0.0181 | 0.14801 | 0.02482 | 0.14781 | 0.01371 | 0.17445 | 0.00595 | 0.13173 |  | - | - | - | + |
| Gambia | 0.01654 | 0.21746 | 0.08175 | 0.01558 | 0.03792 | 0.00838 | 0.03318 | 0.03 | 0.05019 | 0.01226 | 0.1563 | 0.00836 | 0.09141 | 0.00747 | 0.11217 | 0.01037 | 0.12354 | 0.02139 |  | - | - | + |
| China | 0.10225 | **0.44783** | 0.22386 | 0.10203 | 0.09687 | 0.09341 | 0.17593 | 0.1862 | 0.19416 | 0.09836 | 0.2209 | 0.12734 | 0.07728 | 0.06387 | 0.27225 | 0.10154 | 0.20243 | 0.09801 | 0.09489 |  | - | + |
| Benin | 0.0316 | 0.32996 | 0.08844 | 0.02927 | 0.05098 | 0.03291 | 0.06073 | 0.0714 | 0.10367 | 0.03441 | 0.24531 | 0.05455 | 0.05866 | 0.01588 | 0.11011 | 0.03761 | 0.14961 | 0.04353 | 0.01818 | 0.08014 |  | + |
| India | 0.05351 | 0.16519 | 0.08623 | 0.04526 | 0.04221 | 0.05121 | 0.05053 | 0.0476 | 0.02824 | 0.03773 | 0.11131 | 0.03564 | 0.14921 | 0.04313 | 0.12947 | 0.04164 | 0.15053 | 0.04317 | 0.03983 | 0.1485 | 0.07652 |  |

Colour coding: High: 0.15-0.25 Moderate: 0.05-0.15 Low: 0-0.05 Negative
